# 3D-CTBA结合灌注区识别技术在单孔胸腔镜复杂肺段切除术中的应用

**DOI:** 10.3779/j.issn.1009-3419.2023.101.02

**Published:** 2023-01-20

**Authors:** Yuanbo LI, Yi ZHANG, Xiuyi ZHI, Lei SU, Baodong LIU

**Affiliations:** 100053 北京，首都医科大学宣武医院胸外科; Department of Thoracic Surgery, Xuanwu Hospital, Capital Medical University, Beijing 100053, China

**Keywords:** 肺肿瘤, 单孔胸腔镜, 肺段切除术, 段间界, 灌注区, 识别技术, Lung neoplasms, Uniport thoracoscopy, Segmentectomy, Intersegmental plane, Perfusion area, Identification technique

## Abstract

**背景与目的:**

随着肺段切除术在早期肺癌治疗中的广泛应用，如何更加精准和微创地完成肺段切除成为研究热点。本研究旨在探讨三维计算机断层支气管血管成像（three-dimensional computed tomography bronchography and angiography, 3D-CTBA）结合灌注区识别技术在单孔胸腔镜复杂肺段切除术中的应用价值。

**方法:**

回顾性分析2021年1月-2022年1月首都医科大学宣武医院胸外科单诊疗组连续112例单孔胸腔镜复杂肺段切除术患者的资料，总结采用三维重建结合灌注区识别技术完成手术并分析其临床数据。

**结果:**

本组病例平均手术时间为（141.1±35.4）min，段间界显示初始时间为（12.5±1.7）s，段间界维持时间为（114.3±10.9）s，肺段间界均清晰显示（100%），出血量为[10 (10, 20)]mL，术后总引流量为（380.5±139.7）mL，术后拔管时间为（3.9±1.2）d，术后住院时间为（5.2±1.6）d。8例出现术后并发症。

**结论:**

3D-CTBA结合灌注区识别技术在单孔胸腔镜复杂肺段切除术中对段间界识别具有快速、准确和安全的优点，为精准切除肿瘤、减少手术时间及降低手术并发症提供指导。

## Abstract (en)

**Background and objective:**

With the extensive application of segmental lung resection in the treatment of early-stage lung cancer, how to complete segmentectomy more accurately and minimally invasively has become a research hotspot. The aim of this study is to explore the application of three-dimensional computed tomography bronchography and angiography (3D-CTBA) combined with perfusion area recognition technique in single-hole thoracoscopic complex segmentectomy.

**Methods:**

From January 2021 to January 2022, the clinical data of 112 consecutive patients undergoing single-port thoracoscopic complex segmentectomy in the Department of Thoracic Surgery, Xuanwu Hospital, Capital Medical University were retrospectively analyzed. The three-dimensional reconstruction combined with perfusion area identification technique was used to perform the operation and the clinical data were analyzed.

**Results:**

The average operation time was (141.1±35.4) min; the initial time of intersegmental plane display was (12.5±1.7) s; the maintenance time of intersegmental plane was (114.3±10.9) s; the intersegmental plane was clearly displayed (100%); the amount of bleeding was [10 (10, 20)] mL; the total postoperative drainage volume was (380.5±139.7) mL; the postoperative extubation time was (3.9±1.2) d; and the postoperative hospitalization time was (5.2±1.6) d. Postoperative complications occurred in 8 cases.

**Conclusion:**

The advantages of 3D-CTBA combined with perfusion area recognition technique are fast, accurate and safe in identifying intersegmental boundary in single-port thoracoscopic complex segmentectomy, which could provide guidances for accuratding resection of tumors, shortening operation time and reducing surgical complications.

近年来，随着胸部计算机断层扫描（computed tomography, CT）筛查的普及，越来越多的肺结节被早期发现。其中磨玻璃结节（ground-glass nodule, GGN）成为胸外科医生日常面临的主要疾病之一^[1]^。JCOG0802/WJOG4607L研究^[2]^肯定了肺段切除在早期肺癌中的可行性和安全性，通过肺段切除不但完整切除肿瘤而且保留了更多的肺功能。而在肺段切除术中，其靶段段门结构的解剖变异以及段间界的精准识别尤为重要，特别是针对复杂肺段切除术^[3]^。通过三维计算机断层支气管血管成像（three-dimensional computed tomography bronchography and angiography, 3D-CTBA）可了解段门结构的解剖变异，而段间界的主要识别方法有改良膨肺萎陷法和荧光反染法等，但前者作为通气区识别技术存在术中等待段间界出现的时间长，在慢性阻塞性肺疾病（chronic obstructive pulmonary disease, COPD）患者中尤为明显，并且正压通气膨肺可能造成肺损伤、靶段肺组织因充气而取出困难以及上述因素可能导致的气腔播散（spread through air spaces, STAS）和触摸寻找结节不便等。后者作为灌注区识别技术能够更快速、更准确地识别段间界^[4]^。因此，如何在单孔胸腔镜复杂肺段切除术中精准地切除肿瘤、减少手术时间及降低手术并发症，本研究利用通气区-灌注区的概念回顾性总结了3D-CTBA结合荧光反染法在单孔胸腔镜复杂肺段切除术中的应用价值。

## 1 资料与方法

### 1.1 一般资料

回顾性分析首都医科大学宣武医院胸外科单诊疗组2021年1月-2022年1月连续112例单孔胸腔镜复杂肺段切除术患者（排除了左上叶舌段、固有段及左右下叶背段）的资料。其中所有患者均完成术前强化CT。入组标准：结节直径≥8 mm且≤2 cm，位于肺中外1/3需要行肺段切除术加肺门和纵隔淋巴结清扫，必要时进行术中冷冻病理诊断或细胞学检查，以确认切缘没有肿瘤细胞、淋巴结是否转移，还可以鉴定肿瘤病理亚型（包括微乳头、实性成分以及STAS），当出现以上高危因素时，应进行扩大切除。其中男性40例，女性72例，平均年龄为（58.00±11.41）岁，术前合并高血压27例，II型糖尿病16例，COPD 8例。肺功能平均第一秒用力呼气量（forced expiratory volume in the first second, FEV_1_）为（2.49±0.65）L，FEV_1_%为（78.71±8.43）%。手术部位如表1所示，影像学肿瘤平均直径为（1.08±0.34）cm，其中纯GGN（pure GGN, pGGN）42例，混合型GGN（mixed GGN, mGGN）70例。术前患者完成血常规、生化、凝血、肿瘤标志物、心电图和肺功能检测，美国东部肿瘤协作组（Eastern Cooperative Oncology Group, ECOG）评分等无手术禁忌证。本研究经首都医科大学宣武医院伦理委员会批准（批准号：KS2022141）。

**表1:** 单孔胸腔镜复杂肺段切除的手术部位

Item	n
Right side	65
S1	16
S1+S3b	2
S2	13
S2+S1a	5
S3	9
S2b+S3a	2
S7+S8	3
S8	7
S9+S10	4
S10	4
Left side	47
S1+S2	14
S1+S2c	3
S1+S2c+S4	1
S3	8
S7+S8	9
S8+S9	2
S9+S10	4
S10	6

### 1.2 术前3D-CTBA

将术前胸部强化CT的影像资料以DCIOM格式0.625 mm层厚导入Mimics软件，自动重建支气管结构，对于亚段支气管成像不全时需手工延长支气管结构。标记靶病灶大小及位置，模拟病灶完成结节重建。虚拟重建血管图像，并自动区分肺动脉及静脉，由于段及以下水平血管错综交汇，电脑软件对与局部肺动脉和静脉有自动识别错误的情况发生，需结合解剖知识手动更改相应动脉及肺静脉走行。根据3D-CTBA结果，明确靶段相应支气管及血管，规划手术方案（图1A）。对于直径<10 mm且距离胸膜>15 mm的pGGN常规术前使用化学胶定位，以便术中更快捷准确地找到结节位置。

**图1:**
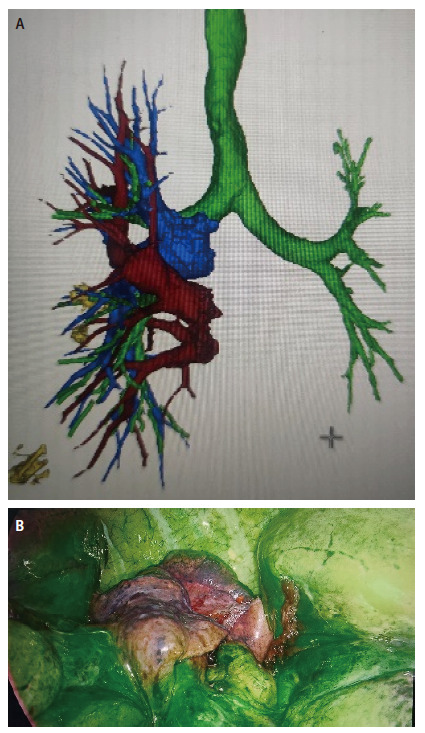
三维重建与灌注区域识别示例。A：利用Mimics软件重建支气管、肺结节和肺动静脉；B：外周静脉注射吲哚晶绿后使用荧光胸腔镜显示段平面。

### 1.3 手术流程

全麻双腔气管插管，取腋前线第4肋间（上叶）或第5肋间（下叶）做3 cm切口。肺萎陷后，根据术前化学胶定位在其肺表面电钩烧灼或者缝线标记，然后手术探查选择纵隔胸膜、叶间裂或者下肺韧带入路。按照术前3D-CTBA的规划，仔细辨认段门结构。对于上叶前段手术，则一般先处理靶段静脉，而后处理靶动脉。游离段动脉时打开动脉鞘，尽量向远端游离，仔细辨别靶段动脉并做切断处理（较粗的动脉用缝合器械处理，较细的动脉可近端丝线结扎远端超声刀处理）。利用靶段支气管与同靶段动脉伴行这一解剖学特点，在分离靶段支气管后，缝合器夹闭靶段支气管。嘱麻醉医师以正压膨肺（不超过20 cmH_2_O），待保留段肺组织复张后即停止正压通气改为单肺通气，明确病灶位于萎陷的靶段内后离断缝合靶段支气管。提起支气管残端，处理段内静脉，但对于段间静脉应尽量保留以避免术后咯血。采用荧光反染法明确靶段动脉灌注缺失区域范围，巡回护士将吲哚菁绿（indocyanine green, ICG）25 mg溶于10 mL生理盐水，经过外周静脉快速注射，助手同时将红外线胸腔镜（infrared thoracoscopy, IRT）打开荧光模式，含有ICG的保留肺组织吸收805 nm的光，反射940 nm的光，在IRT下呈蓝色；而靶段不含ICG，呈白色，以此显示段间界，电钩于肺脏层胸膜表面灼烧标记段间界后退出荧光模式（图1B）。超声刀尽量提升段门结构，使用直线切割缝合器沿着此前的标记点适形裁剪段间界，保证保留段肺组织舒展，切除肺段放入标本袋中经切口取出。剖开标本，找到病变，缝线标记送冰冻病理。对于浸润性腺癌（invasive adenocarcinoma, IA），需常规送检N1组淋巴结至快速病理，如为阳性则转做肺叶切除+淋巴结清扫。对于原位癌及微浸润腺癌（minimally invasive adenocarcinoma, MIA），常规做淋巴结采样。术后于切口放置20#胸腔闭式引流管1根。

术后常规应用预防性抗生素48 h-72 h，同时雾化排痰并鼓励患者早期下床活动。胸腔闭式引流拔管指征：胸腔引流<150 mL/d，咳嗽无漏气且复查胸片肺复张良好。

### 1.4 观察指标

对患者手术时间、出血量、段间界显示时间和维持时间、引流量、拔管时间、术后住院时间及剪裁段间界切割缝合器使用数量、病理学类型[不典型腺瘤样增生（atypical adenomatous hyperplasia, AAH）、原位腺癌（adenocarcinoma in situ, AIS）、MIA和IA]、清扫淋巴结数量、病灶至切缘距离及术后并发症情况进行记录。

### 1.5 统计学方法

应用SPSS 23.0进行数据分析。符合正态分布的计量资料以均数±标准差（Mean±SD）表示。不符合正态分布的计量资料以中位数（下四分位数，上四分位数）[M (P25, P75)]表示。

## 2 结果

全组患者均顺利完成手术，无围术期死亡，无中转开胸和增加操作孔。手术时间为（141.1±35.4）min，段间界显示初始时间为（12.5±1.7）s，段间界维持时间为（114.3±10.9）s，均在荧光染色弥散前顺利完成荧光物质高低灌注区界限的标记。手术出血量为[20)]mL，术后总引流量为（380.5±139.7）mL，剪裁段间界切割缝合器使用数量为（3.6±0.8）个。术后病理分型：AAH 4例，AIS 47例，MIA 32例，IA 29例，清扫淋巴结数量为（5.4±2.5）枚，病灶至切缘距离为（2.3±0.6）cm。

术后并发症8例（7%）。其中2例肺部感染，经痰培养药敏培养指导下升级抗生素后治愈；2例持续漏气时间超过7 d，分别采用缓冲负压吸引，或者胸腔内灌注50%高张葡萄糖50 mL治疗后好转，分别于术后10 d和12 d拔管；2例房颤，给予胺碘酮静脉微量泵治疗后复律；1例肺不张，予气管镜吸痰后改善，胸片示肺复张良好；1例肝功能异常，术后第3天谷丙转氨酶由术前11 IU/L升至251 IU/L，谷草转氨酶由17 IU/L升至214 IU/L，考虑药物性肝损伤，予还原型谷胱甘肽和多烯磷脂酰胆碱药物对症治疗，1周后转氨酶恢复正常。本组病例术后拔管时间为（3.9±1.2）d，术后住院时间为（5.2±1.6）d。

## 3 讨论

随着麻醉技术的完善、手术器械的改进、手术技巧的提高和围术期管理的进步，肺癌诊治朝着安全性、精准性和微创性的方向发展。“微创”理念是指某种技术的创口（外观创伤）最小和对全身（应激反应）影响最小（两个最小），“精准”理念是指某种技术对病灶（内在创伤）最大范围地获取或清除和对脏器最大限度地保护（两个最大）^[5]^。近年来，有研究^[6]^证实肺段切除术已经成为早期肺癌的标准治疗方式，与肺叶切除术相比，在复发率和5年生存率上无差异，同时还可减少并发症和死亡率，有利于肺功能的保护。尤其是胸腔镜手术的广泛开展，给广大患者带来了更微创的治疗选择。

肺段切除术的最佳适应证是病灶位于段或亚段中心。然而临床上病灶的位置常常不位于段中心或亚段中心，因此肺段切除术的方式除了单一肺段切除以外，还可能存在肺段+亚段切除、肺段扩大切除、单一亚段切除和联合亚段切除等术式。临床上还常将肺段切除术根据段间界的交界面数量或角度分为简单肺段切除术（包括左肺上叶固有段和舌段以及双下肺背段）和复杂肺段切除术（其余肺段）。除此以外，段门结构的解剖变异广泛存在，因此术前了解靶段段门结构的解剖变异以及术中段间界的精准识别尤为重要^[7,8]^。我中心术前常规采用Mimics软件进行3D-CTBA，可以准确判断结节所在的靶段，了解靶段血管及支气管的解剖结构和相互关系，判断是否存在畸形和变异，从而避免误伤靶段血管和支气管。此外，我们在术中处理靶段血管以后，游离出靶段支气管并用切割缝合器夹闭而不激发，嘱麻醉医生膨肺，确保保留段肺组织复张即可，如果病灶标记位置位于萎陷的靶段内，再击发切割缝合器，切断靶段支气管，因为靶段支气管的处理是肺段切除术的核心操作；另外，如果按照改良膨胀-萎陷法识别段间界，可能会造成肺损伤、靶段肺组织因充气而取出困难以及上述因素可能导致的STAS和触摸寻找结节不便等。

段分界是相邻肺段之间的一层薄膜，它的处理是复杂肺段切除术的重点和难点，主要是因为它的多维性。肺段切除术中如未能准确识别段间界，会影响切除范围的准确性，从而造成切缘不足、切除过多、段间静脉损伤、术后咯血等。段间界的识别技术是基于靶段和保留段肺组织的通气区差异和灌注区差异。前者通过靶段支气管辨别段间界，如改良膨胀-萎陷法，即靶段支气管切断后纯氧膨肺，由于Kohn’s孔的存在，靶段的肺组织也会膨胀；在整个肺组织膨胀后恢复单肺通气，保留段肺组织会萎陷，而靶段肺组织需要10 min-15 min才有可能萎陷，使得充气靶段和萎陷的保留段肺组织之间形成分界线，也就是段间界^[9]^。该方法的优点是段间界维持时间长，缺点是等待时间长，尤其是对于COPD患者，因为肺的颜色较深，肺顺应性下降，使得膨胀-萎陷相对困难，延长了段间界成型时间，或者难以成型，从而进一步延长了手术时间。此外，膨起的靶段肺组织更难经过切口从体内取出，标本容易碎裂甚至导致STAS，甚至有的患者会单纯因为取标本而延长切口造成切口痛。还有学者^[10]^提出肺循环单向阻断方法，它利用靶段动脉或静脉阻断后膨肺，因血流灌注缺失的区域造成局部氧不能排出，因而表现为鲜红色，非靶段因血流循环完整而体现为乏氧的暗红色。通过色差确认段间界，但该方法应用在北方采暖区或者长期大量吸烟的患者中，会由于肺气肿和碳墨沉积等造成肺表面颜色难以仅通过血流含氧量高低的色差来辨别。

根据既往文献^[11]^报道，基于段动脉切断后灌注区差异的荧光反染法较膨胀-萎陷法在段间界识别上更精确，可能是由于前者存在Kohn’s孔的原因。在靶段动脉切断后，静脉注射的ICG无法进入靶段，使用IRT荧光模式下确定段间界，通常会在10 s左右完成显像，并清晰地显示灌注缺损区的准确范围。本研究中显示初始时间为（12.5±1.7）s，但由于存在ICG弥散问题，段间界持续时间约2 min，需要用电钩快速在脏层胸膜表面标记出段间界界限，本组病例均顺利完成段间界的标记。

本研究中，3D-CTBA可以明确结节的位置及段门结构血管，指导术中准确解剖切断段血管和支气管，阻断靶段动脉的灌注区和靶段支气管的通气区，再结合荧光反染法能快速准确地识别该灌注缺失区和通气缺失区，使切除范围更精确。本组病灶至切缘距离为（2.3±0.6）cm，术后病理无距离切缘过近病例。在足够的切缘基础上由于切割缝合器向萎陷区（通气缺失区）的滑滚作用在理论上保留了更多的肺组织，简化了操作流程。

本组病例无围术期死亡，无中转开胸和增加操作孔。术后无严重并发症和术后咯血。8例出现并发症患者中漏气2例（1.7%），发生率低，均为长期吸烟的慢性肺气肿患者，且胸腔内存在广泛粘连。在分离粘连和段间组织中，电钩和超声刀的应用可能对肺质量差的患者造成的肺组织损伤更高，术后漏气也延长了患者的术后拔管时间。我们通过缓冲负压吸引和胸腔内注射高张葡萄糖的方法顺利治疗了漏气的患者。

随着胸腔镜肺段切除术的普及和应用，从术前规划到术中段门的解剖和段间界的识别，都给胸外科医生带来更多要求和挑战。本研究中3D-CTBA结合灌注区识别技术在复杂肺段切除术中对段间界识别上具有快速、准确和安全的优点，为精准切除肿瘤、减少手术时间及降低手术并发症提供指导，值得在临床推广。但是其对肺功能的长期影响和是否存在局限性肺不张的情况则需要进一步临床研究加以证实。
